# Error in Affiliation

**DOI:** 10.1001/jamanetworkopen.2026.43457

**Published:** 2026-10-16

In the Original Investigation titled “Clinical Digital Health Engagement and Psychological Distress Among Adults,”^1^ published July 16, 2026, there was an error in the affiliation for Dr S. O. Onal. This article has been corrected.^1^
